# Automatic thresholding from the gradients of region boundaries

**DOI:** 10.1111/jmi.12474

**Published:** 2016-09-20

**Authors:** G. LANDINI, D.A. RANDELL, S. FOUAD, A. GALTON

**Affiliations:** ^1^ School of Dentistry University of Birmingham Birmingham U.K.; ^2^ Department of Computer Science University of Exeter Exeter U.K.

**Keywords:** Image processing, mathematical morphology, segmentation

## Abstract

We present an approach for automatic threshold segmentation of greyscale images. The procedure is inspired by a reinterpretation of the strategy observed in human operators when adjusting thresholds manually and interactively by means of ‘slider’ controls. The approach translates into two methods. The first one is suitable for single or multiple global thresholds to be applied globally to images and consists of searching for a threshold value that generates a phase whose boundary coincides with the largest gradients in the original image. The second method is a variation, implemented to operate on the discrete connected components of the thresholded phase (i.e. the binary regions) independently. Consequently, this becomes an adaptive local threshold procedure, which operates relative to regions, rather than to local image subsets as is the case in most local thresholding methods previously published. Adding constraints for specifying certain classes of expected objects in the images can improve the output of the method over the traditional ‘segmenting first, then classify’ approach.

## Lay description

Extracting information from digital images often requires applying a process called ‘segmentation’ which consists of grouping picture elements (pixels) into regions that correspond to the features or objects of interest depicted in an image. Among the many approaches to segmentation, greyscale ‘thresholding’ is used to assign to pixels “object” (or foreground) or “non‐object” (background) labels, depending on their intensity value relative to a reference value called ‘threshold’ which can be specified manually or automatically. We reinterpret a previously published observation of how humans search manually for threshold values when using ‘slider’ controls to adjust threshold values by trial and error, and use this to implement two methods of automatic thresholding. The first method (suitable for determining one or several global thresholds) consists of searching for the greyscale value on the image that generates a phase (the set of all pixels potentially labelled as “object”) whose boundary with the foreground coincides with the largest gradients (changes of intensity between a pixel and its neighbours) in the image. The second method involves further modifications that consider all regions in the phase independently rather than as a whole. This results in the method becoming an adaptive, local threshold procedure, which is based on the analysis of the regions, rather than on local image subsets as is the case with most local thresholding methods published in the literature. This new regional thresholding approach can include constraints for specifying certain classes of expected objects in images to improve the output over the traditional approach where an image is segmented first and then filtered for regions that match a given specification. The gain from this approach arises from the ability of the method to consider all possible regions that could be generated at various thresholds and retain only those that match the specifications and the image gradients, instead of retaining those from a fixed, perhaps not optimal initial threshold.

## Introduction

An important aspect to consider in imaging‐based quantitative microscopy is to be clear about what processed images represent and to make explicit as far as is practically possible the assumptions the processing algorithms used. Here we deal with procedures to group image sample points into labelled regions, which are generically referred to as *segmentation* techniques. Within those, *thresholding* is the labelling of picture elements (pixels) typically, although not exclusively, into two phases, one forming regions that represent the objects of interest as foreground and the other, non‐object regions as background. Such approaches commonly use the distribution of pixel values in the image to find a value (the threshold) that determines the limit between the phases in the measurement space (typically representing a quantity such as light absorbance, reflectance or emission). Although numerous thresholding methods have been proposed (Sezgin & Sankur, 2004), none are generic enough to be successful when applied unsupervised to arbitrary images with different characteristics; consequently, there is still a need to search for better thresholding algorithms.

Thresholds can be computed globally for a whole image (see e.g. Sezgin & Sankur, 2004 for a review) or locally, breaking down the analysis into image subsets, or local windows. Local thresholds are useful, for example, when image illumination is not homogeneous and a single threshold value does not yield acceptable results. Numerous thresholding methods are based on features obtained from the image intensity histogram (e.g. Otsu, 1979); this has the advantage that the threshold value is found by operating on a one‐dimensional array. Operating on the histogram is convenient as it is usually orders of magnitude smaller than the image data itself and therefore opens in some cases the possibility of nearly real time imaging applications, where the processing of successive frames requires the computation to be done fast. However, not all imaging requires real‐time processing; for example, in many microscopy applications specimens are fixed and scenes do not change with time, and therefore better performing methods, even if slower, might be preferable.

Here we present first a simple procedure to find optimal global threshold values on images containing objects with greyscale values distinct from that of the background. We then introduce a variation of the method that enables the identification of objects with specific morphological characteristics independent of a global threshold. This modified method can be interpreted as a local thresholding that is *region*‐based, rather than *local set*‐based. Examples are given for nuclear segmentation in cultures of H400 cells (an oral carcinoma cell line) grown on glass cover‐slips and stained with haematoxylin.

## Materials and methods

The conventions used in this paper are as follows. ‘Phase’ is the set of all pixels labelled as foreground at some tentative threshold value. ‘Regions’ are sets of pixels belonging to the phase that are locally connected under 8‐neighbours connectivity, whereas ‘objects’ are those regions that correspond to ideally segmented ontologies. Consequently, the phase contains all regions at a given threshold and the aim is to find a threshold value that matches the regions as closely as possible to the objects represented in the image. It is important to note that pixels, regions, and objects characterise distinct stages in the passage from biological objects in reality to our reconstructed models of them. In Galton *et al*. (2016) we associate each of these stages with a distinct ontological level; in terms of the scheme described there, the operations considered in the present paper are concerned with the transition from Level 1 (captured images as pixel arrays) to Level 2 (segmented images comprising candidates for consideration as model objects).

### Global thresholding

The rationale of the procedure is based on an early observation of how individuals appear to intuitively determine image threshold values interactively when using imaging applications (e.g. when manipulating a slider controlling the global threshold value). Users tend to repeatedly adjust the value (over‐ and undersegmenting the image) until the phase more or less ‘matches’ the objects. This strategy was noticed by Russ & Russ (1987) and published originally in this journal. The authors reported that when an optimal threshold value is found, the shape of the phase representing the objects tends to become stable across a range of threshold values, and the length of the segmented object perimeters is also stable. They further suggested searching for such a plateau over a range of threshold values. However, it is not straightforward to deal with multiple objects to account for more than one plateau in the distribution of single object perimeters, or for varying the number of regions at different threshold levels.

Here we propose an alternative and convenient interpretation of Russ and Russ's original observation: namely, that when an object is optimally thresholded, the *boundary of the thresholded phase* should, by definition, coincide with the *boundary of the objects* in the original image. Given that figure‐objects and background in an image have different intensities, the edge of the optimally thresholded phase should also coincide with large image gradient values in the original image. Therefore, finding the optimal threshold is equivalent to searching for the greyscale level at which the sum of image gradients occurring at the edge of the phase is maximised. Although this could be considered a simple and obvious approach, it does not appear to have been described before in algorithmic terms. More importantly, it provided the basis to develop a further, more robust and accurate region‐based thresholding method, which is discussed later.

### Implementation details

The algorithm is relatively straightforward and can be implemented in any standard imaging platform that enables access to image pixel values directly. In our case, all procedures were implemented by us using the popular ImageJ version 1.51 platform by Rasband (1997).

In the following examples we assume an image with dark objects (as foreground) on a bright background to specify the search, but the procedure can be applied in the inverse case with a simple modification described later.

The method can be summarised as follows: from the input greyscale image *I*, a gradient image *G* is first computed, where the gradient magnitude at each pixel *p*(*x, y*) of *I* is represented as pixel intensity. A variety of established methods exist to compute this, for example, convolution filters (Laplacian, Sobel, Prewitt, Kirsch, Frei and Chen) (Russ, 1999) as well as morphological methods, such as greyscale morphological gradients (Beucher, 1990; Rivest *et al*., 1993).

The optimal global threshold *T*_Optimal_ is then found by means of an exhaustive search through all possible threshold levels *L* (i.e. between the grey levels *L*_Min_ and *L*_Max_) of *I*. First, a binary image *B*(*L*) is generated by setting a test threshold of *I* in the range from *L*_Min_ to *L* (or from *L*_Max_ to *L* when searching for bright objects on a dark background). From *B*(*L*), another binary image *E*(*L*) is computed to represent only the *edges* or *boundary of the thresholded phase* of *B*(*L*). Again, various options exist to generate this binary boundary, two practical procedures are: (a) the Laplacian convolution filter approximated with kernels such as

0−10−14−10−10 or −1−1−1−18−1−1−1−1,

and (b) the *internal morphological gradient* of *B*(*L*) computed as the difference between the original and the morphological erosion of the original, that is,

(1) E(L)=B(L)−(B(L)⊖A),

where *A* is a 3 × 3 pixel structuring element and **⊖** is the morphological erosion operation.

The number of pixels *N*(*L*) forming the thresholded phase boundary in image *E*(*L*) is also counted (to be used later in Eq. (3)). The final step consists of computing the total gradient *G*_Total_ (*L*) (in image *G*) for all the phase boundary pixels *p* in *E*(*L*).

(2) G Total L=∑p∈ELG(p),

where *G*(*p*) is the value of the gradient at point *p*, and finally set *T*_Optimal_ to the *L* value that maximises *G*_Total_ (*L*).

**Figure 1:**
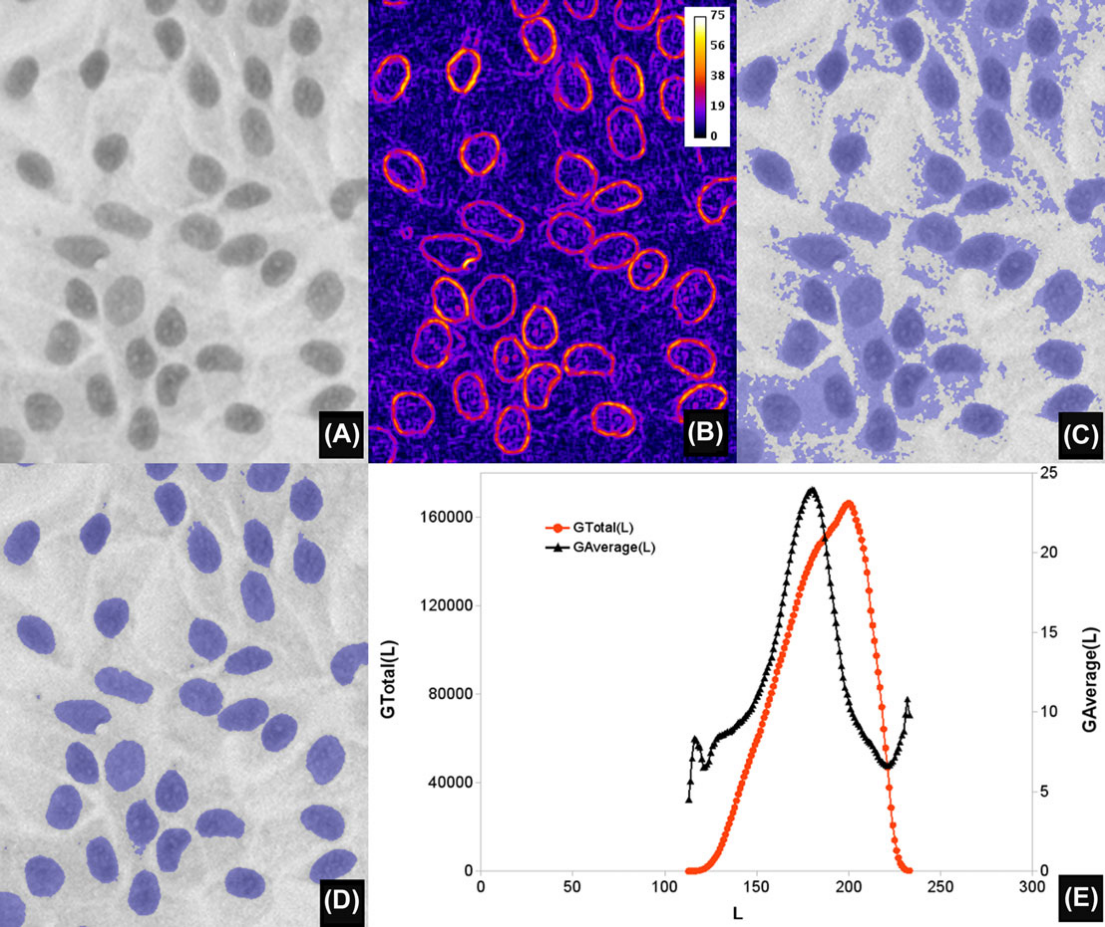
Automated global thresholding. (A) A greyscale haematoxylin stained image of a monolayer of H400 keratinocytes cell line, grown on glass (magnification ×20, field width 168 μm), preprocessed with a Gaussian filter of radius 1. (B) Image gradient of (A), computed with the Beucher morphological gradient and displayed as pseudo‐colour, for clarity purposes. (C) Thresholded (overlay in blue) image obtained using the value *L* at which *G*_Total_ (*L*) is a maximum (*L* = 200). (D) Result of using the value *L* at which *G*_Average_ (*L*) shows a maximum (*L* = 180). Note how, in this particular image, (D) corresponds faithfully to the extent of the nuclei depicted in (A). (E) Plots of *G*_Total_ (*L*) and *G*_Average_ (*L*) as a function of threshold *L* for (A).

**Figure 2:**
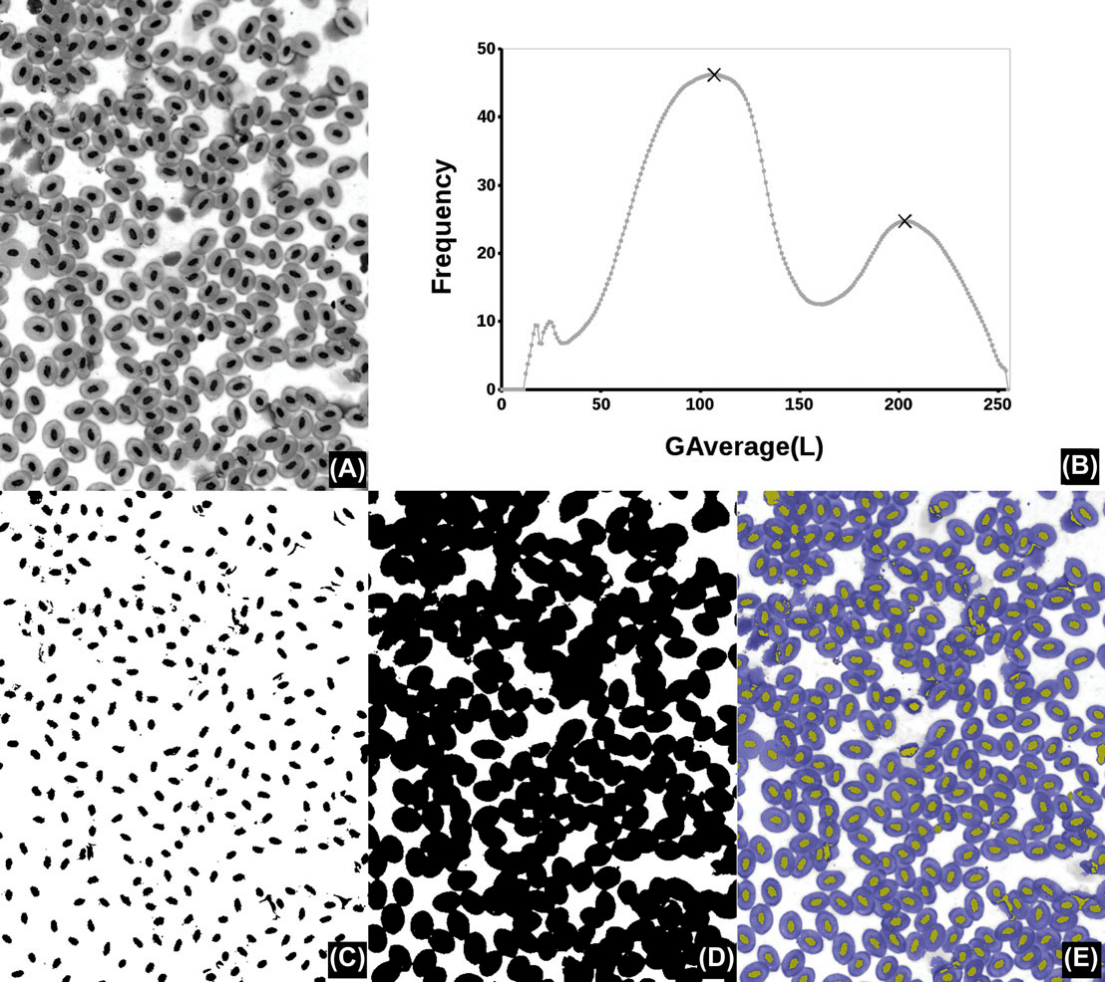
Automated global thresholding example. (A) A frog's blood smear (Giemsa stain, magnification ×40, field width 354 μm). (B) Graph of *G*_Average_(*L*) as a function of the threshold *L* featuring two main maxima (at *L* = 107 and 203, marked with “Х”). (C), (D) Binary result of (A) thresholded at those maxima values (0..*L* shown in black), which coincide with cells and nuclei respectively. (D) Results as overlays on the original (nuclei in yellow, cells in blue).

Alternatively, another measure to find the optimal global threshold is the *average gradient at the phase boundary*:

(3) G Average L=∑p∈ELG(p)NL,

The plot of *G*_Total_(*L*) and *G*_Average_(*L*) versus *L*, in the test images investigated, typically has a global maximum that makes it easy to determine *T*_Optimal_. A pseudo‐code description of the algorithm is shown in Table 1, and examples are shown in Figures 1 and 2.

**Table 1:** Pseudo‐code for the global threshold algorithm using the gradient of region boundaries.

Find minimum *L*_Min_ and maximum *L*_Max_ grey levels of image *I*
Compute image gradient (Sobel filter or Beucher gradient *G =* (*I* ⊕ *A*) – (*I* **⊖** *A*))
For all values *L* from *L*_Min_ to *L*_Max_ {
Set a test threshold at *L*
Create a thresholded phase *B*(*L*) by labelling pixels valued 0 to *L* as foreground
Compute the phase boundary *E*(*L*) = *B*(*L*) – (*B*(*L*) **⊖** *A*)
Store the total *G*_Total_(*L*) (or average *G*_Average_(*L*)) gradient (in *G*) of pixels in *E*(*L*)
}
Set *T*_Optimal_ to the *L* with maximum *G*_Total_ (*L*) (or average *G*_Average_ (*L*))

### Features of the gradient plots

In most images we investigated, the plots of *G*_Total_ (*L*) or *G*_Average_(*L*) versus *L* are relatively smooth with a global maximum suggesting the optimal threshold (Fig. 1E). Additional local maxima can sometimes be present, specially in the *G*_Average_(*L*) plots; these maxima correspond to alternative candidates for global threshold or for multiple thresholds. *G*_Average_(*L*) (computed by dividing the total gradient by *N*(*L*)) has the effect of weighing the strength of the gradients with the boundary lengths; this might be a desirable feature to exploit, depending on image contents. Figure 2 shows a case of two maxima in the gradient plots, which give thresholds for cells and nuclei respectively.

### Region‐based thresholding

The adjustment strategy described by Russ & Russ (1987) to set thresholds manually, applies to images where objects have similar greyscale values. When these differ, or in images with uneven illumination, no single threshold value is able to segment all objects. In such cases, adaptive techniques are necessary. The principles discussed above can be modified further to compute optimal thresholds for every image object independently and that is where our method shows its advantages. The first part of the procedure remains the same as for the global approach: the exhaustive search is performed through all possible threshold levels *L*. At each level the image is also binarised and the boundary pixels are extracted, but now we compute *G*_Average_(*L*) (the magnitude of the average gradient from *G*) independently for each connected component *c* (i.e. each region separately) in *E*(*L*). Those *boundaries of binary regions* are then labelled with their individual *G*_Average_ (*L, c*), and stored in a slice of an image stack *S*, where the slice position represents a given *L*. That is, each slice in the stack is an image containing the boundaries of the binary regions *c* detected at level *L*, labelled with the value of *G*_Average_ (*L, c*).

**Figure 3:**
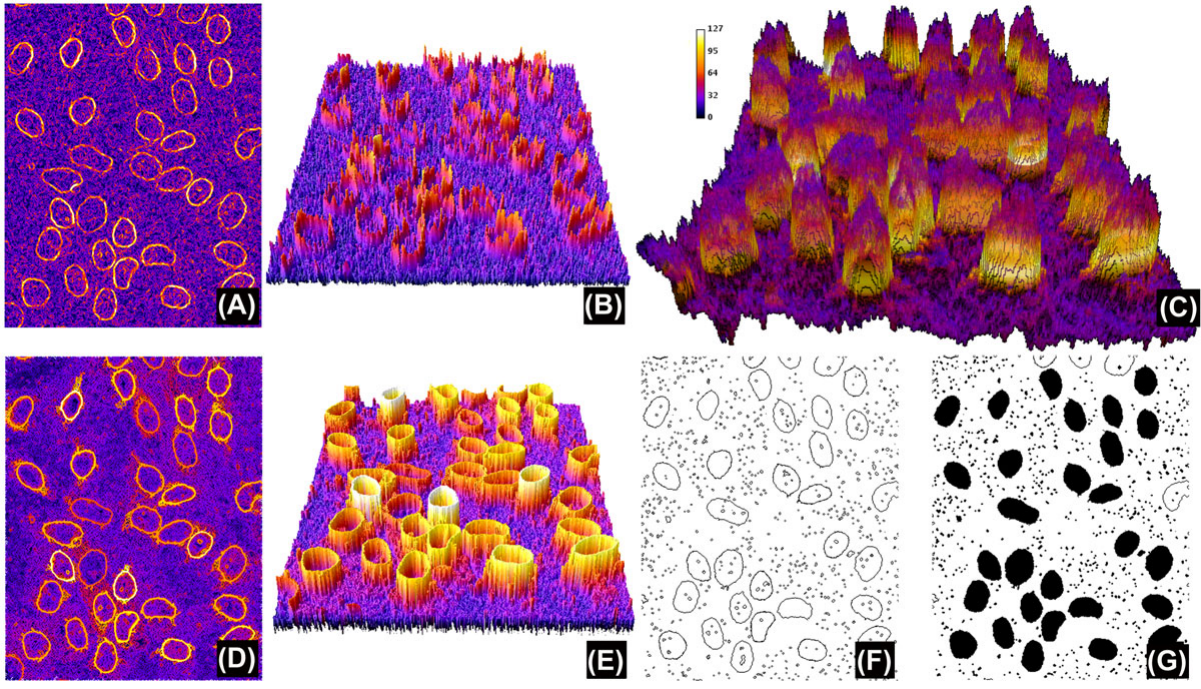
Region‐based segmentation. (A) Gradient of image in Figure 1(A), this time computed using the Sobel filter (note the similarities to Fig. 1B). (B), (E) Pseudo‐3D representation of (A) and (D), respectively. (C) Stack *S*, where the *z* coordinate represents *L* (i.e. the test threshold, the *z* axis inverted for clarity purposes, with high *L* at the bottom). The labelling colour is *G*_Average_ (*L, c*). (D) Maximum projection of stack *S*. Note that, while similar to (A), the intensity has been simplified and the boundaries of regions have all the same value for a given *L* (shown more clearly when comparing (E) with (B)). Computing regional maxima of (D) returns those regions of pixels that are surrounded by strictly lower pixel values (in black, F) and the region filling is shown in (G). Note that this definition of regional maxima means that touching objects could potentially not be detected if they have been labelled with different values (so both cannot be surrounded by strictly lower value pixels). Indeed, three objects in (D) have not been segmented in (F); however, this can be successfully resolved using object specification constraints as shown in Figure 4.

It is worth noting that different objects are often optimally segmented at a different *L* and therefore the detection method becomes relative to each object, unlike the global version of the procedure described earlier. The final task consists of identifying in the stack *S* those region boundaries belonging to objects that have been optimally segmented (i.e. which region and at which *L* it represents an actual object). Although it is expected that the optimally segmented boundaries are labelled with a high value, objects are likely to be included in multiple nonoptimally detected regions generated at other *L*. Finding regions that correspond to optimally segmented objects in the stack data is not straightforward because region boundaries across slices do not necessarily coincide with each other, as they are derived from differing threshold levels. To resolve this, we first compute the maximum projection *P* of the stack, which results in a quantised or simplified version of the image gradient *G* (Fig. 3D). This gradient simplification arises because the boundaries of each region now have homogeneous values (remember they were labelled with the value of *G*_Average_ (*L*, *c*)), instead of containing the variable gradients of image *G* (compare Figs. 3B and D). The pixels forming the boundaries of optimally segmented regions have therefore the highest and homogeneous intensity values in *P*, whereas nonoptimal regions have strictly lower intensity values. The *morphological regional maxima R* (Vincent, 1993) can be then used to identify connected groups of pixels that are surrounded by strictly lower value pixels. These are, finally, the optimally segmented object boundaries, which can be converted to regions by binary region filling in *R*. Pseudo‐code for this method is presented in Table 2 and the most relevant steps shown in Figure 3.

**Table 2:** Pseudocode for the region‐based threshold algorithm using the gradient of region boundaries.

Find minimum *L*_Min_ and maximum *L*_Max_ grey levels of image *I*
Compute image gradient (Sobel filter or Beucher gradient *G =* (*I* ⊕ *A*) – (*I* ⊖ *A*))
For all values *L* from *L*_Min_ to *L*_Max_ {
Set a test threshold at *L*
Create a thresholded phase *B*(*L*) by labelling pixels valued 0 to *L* as foreground
Compute the phase boundary *E*(*L*) = *B*(*L*) – (*B*(*L*) ⊖ *A*)
For each connected component *c* in *E*(*L*) {
Compute the average gradient *G*_Average_ (*L, c*)
Label *c* with *G*_Average_ (*L, c*)
Store the result in slice *L* of image stack *S*
}
}
Compute the Maximum Projection *P* of *S*
Extract the Regional Maxima *R* of *P*
Fill the binary regions in *R*

**Figure 4:**
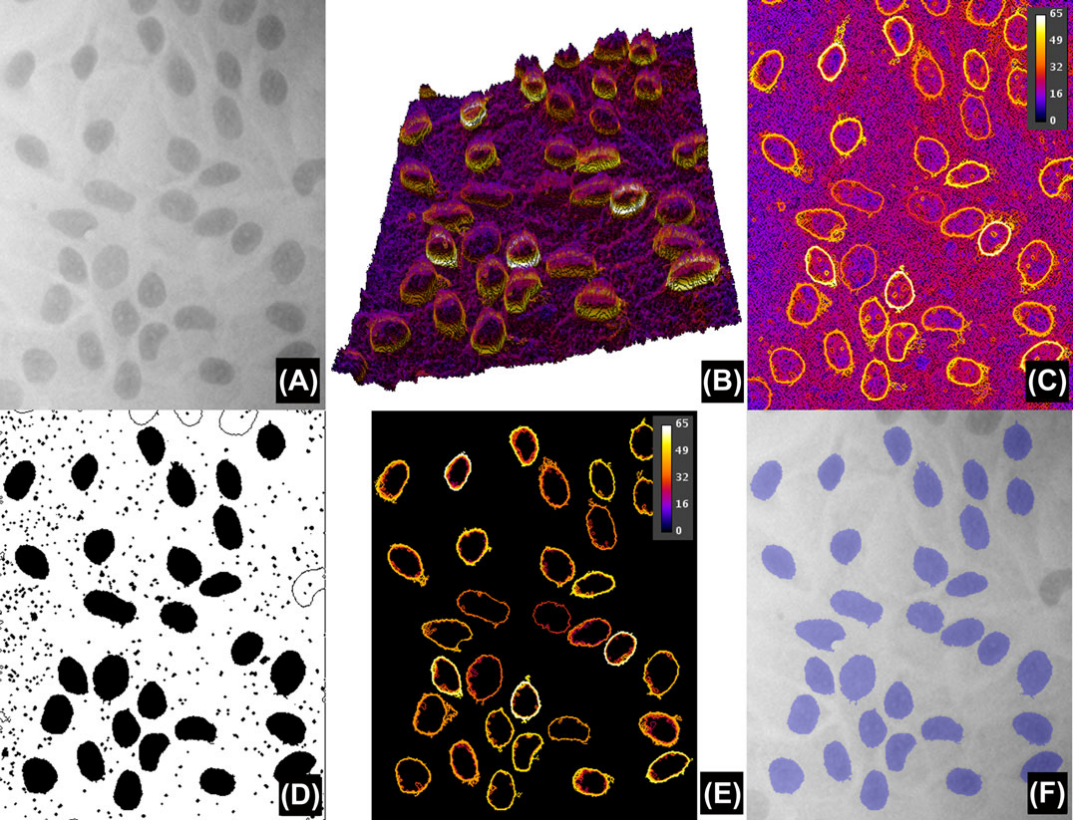
Comparison of results obtained without and with object specification. (A) Version of Figure 1(A) modified to simulate uneven background illumination. (B) Pseudo‐3D projection of the stack *S*, without object specification (the *z* axis inverted for clarity purposes, with high *L* at the bottom). The labelling colour is *G*_Average_ (*L*, *c*) and the tilted slope is due to the difference in local intensity of the image. Compare (B) with Figure 3(C). (C) Maximum projection *P* of stack *S*, whereas (D) is the result after applying the regional maxima and region filling. Note that (C) is very similar to Figure 3(D), in the sense that it recovers a simplified version of the gradients even under uneven backgrounds. (E) Maximum projection *P* of stack *S*, but this time after applying object specification while constructing *S* (in this example, boundaries are only retained if regions have a circularity of at least 0.5 and sizes between 250 and 3000 pixels squared). This results in an even more simplified version of *P* that after the regional maxima and binary filling detects all nuclei in the image (overlay in blue in F).

The procedure described appears at first glance similar to what should be obtained by applying the regional maxima algorithm to the image gradient *G*, but unfortunately this is not so because of the lack of the quantisation of the gradient values in *P* (see Fig. 4).

### Object specification

When objects have a characteristic range of sizes or shapes, segmentation can be significantly improved by applying constraints to the inclusion of region boundaries in *S* only when the regions satisfy conditions such as ‘circularity’ (computed as: 4π Area/Perimeter^2^), minimum and maximum sizes. This results in an even more simplified result *P* (Fig. 4) and makes the application attractive and robust for certain applications, for example, nuclear segmentation. It is also worth noting that the proposed approach is more powerful than the traditional approach using ‘first segmentation followed by classification’ where thresholding is applied, then segmented regions are filtered according to size and shape (discussed later). This is so because although the classic approach relies on the thresholding step to be optimal, the proposed method searches for optimal thresholds (which may vary from region to region) in the data that specify the objects.

**Figure 5:**
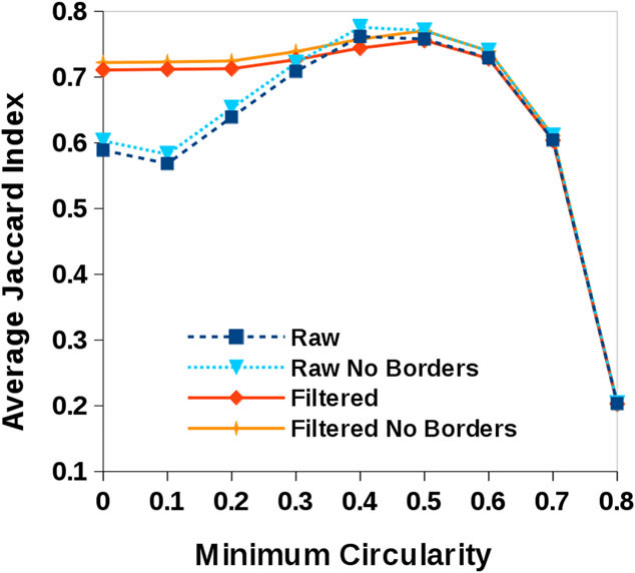
The performance of the region‐based segmentation method proposed depending on the minimum circularity allowed for regions when creating the maximum projection *P* image. ‘Raw’ indicates the output of the segmentation without any further processing, ‘filtered’ means that the output was postprocessed to retain objects with a range of sizes and circularity (as described in the text), finally, ‘no borders’ are the results obtained by removing from the analysis regions that intersect the image frame. The plots indicate that for our images of cell nuclei, minimum circularity values between 0.4 and 0.5 maximise the Jaccard index with respect to the gold standard set containing 1673 objects (1661 objects in the filtered versions of the analysis).

### Evaluation

The region‐based thresholding performance was evaluated against 14 different global and 9 local thresholding methods ported to ImageJ (Landini, 2009 and references therein) from their published descriptions or open source implementations (Niemistö, 2004; Celebi, 2006; J. Bevik, personal communication) to find out how close the various approaches are to an ideal segmentation. First a gold standard set was created by manually segmenting and binarising nuclear profiles in a test set containing a total of 1673 nuclei captured at ×20 magnification (with an interpixel distance of 0.34 μm).

First we investigated the results obtained by our region‐based method according to the minimum circularity value allowed in the object specification. This ranged from 0 (no circularity constraints) to 0.8 (beyond which there were almost no cells detected) in increments of 0.1 and using the range of values of the detected cells in the gold standard image (between 250 and 3000 pixels^2^). The performance of the method was evaluated by means of the Jaccard index (Jaccard, 1901), for objects overlapping between the thresholded and the gold standard images. This index is the ratio of the intersection of two sets to their union, therefore perfectly matching regions between the test and gold standard sets have a Jaccard index of 1, whereas for completely unmatched regions this is 0. Values in between provide a measure of the degree of matching, and the expectation is that good thresholding methods will result in higher Jaccard indexes.

Figure 5 shows the performance of region‐based segmentation method according to the minimum circularity value allowed for regions during the creation of the stack *S* to generate the maximum projection *P* image. The best performance in terms of maximising the average Jaccard index was obtained when populating the stack *S* with boundaries bearing a minimum circularity of between 0.4 and 0.5.

**Figure 6:**
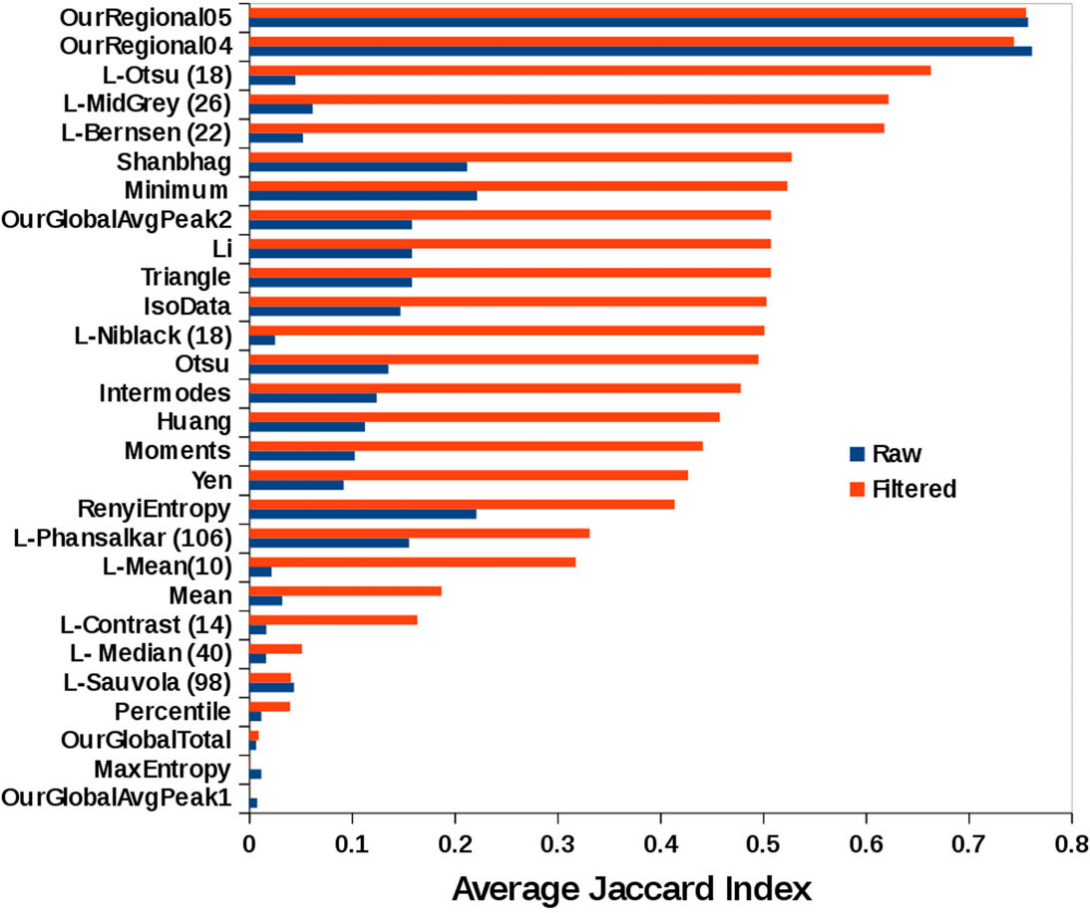
Performance of our segmentation methods in comparison to 23 standard thresholding methods measured by the average Jaccard index (largest is best) against a gold standard set of 1673 hand segmented nuclei. Methods labels preceded by ‘L‐’ are local thresholding methods and the number in brackets is the radius of the local set that produced the highest Jaccard index when comparing to the gold standard. Note that the global based method fails to detect nuclei when setting the threshold to the highest local maximum of *G*_Total_ or *G*_Average_, but it ranks 8th in performance when the second highest local maximum *G*_Average_ is used. Our region‐based method outperformed all other methods investigated in either the raw of filtered versions of the analysis when specifying objects with a minimum circularity values of 0.5 and 0.4 (rows labelled ‘OurRegional05’ and ‘OurRegional04’, respectively).

Second, we examined the performance of our methods against other thresholding procedures. To make the tests comparable, we used a postthreshold filtering step with the same constraints used in our method when it returned the best results (minimum circularity of 0.5 and the size range described earlier), otherwise the number of oversegmented regions returned by several of the other methods was excessively large. For the local threshold methods, we performed an exhaustive search for the optimal size of the local analysis (by setting the radius of the ‘local set’ where the threshold is searched from 10 to 150 in increments of 2) that provided the largest Jaccard index when comparing to the gold standard (these values are shown in brackets in Fig. 6).

Figure 6 shows the average Jaccard index for the three fields analysed by all the tested thresholding methods and compared to the gold standard. Our region method outperformed the other thresholding methods tested.

**Figure 7:**
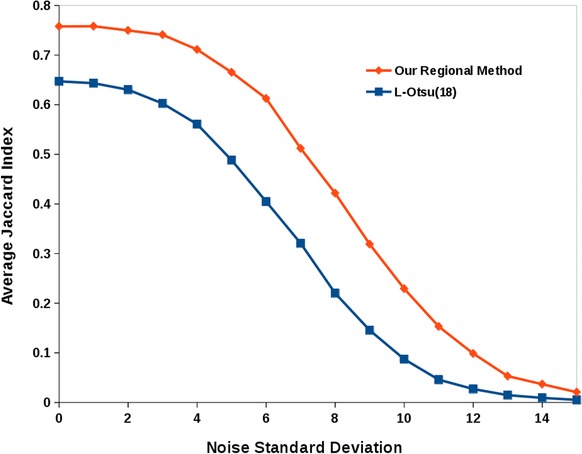
A comparison of the effect of adding pseudo‐random Gaussian noise to the segmentation performance for our region‐based method in comparison to the next best performing method (local Otsu, with a radius of size 18 pixels), both filtered versions of the analysis.

Furthermore, the effect of noise on the measures of segmentation performance (on the object filtered version of the segmentation) was investigated by computing the average Jaccard index obtained after the addition of pseudo‐random Gaussian noise with increasing standard deviations (from 1 to 15 greyscale values). For the purpose of comparison, this was repeated for the second best performing thresholding method (local Otsu, with a radius of 18 pixels). Figure 7 shows that although, unsurprisingly, there is a decrease in the performance of the segmentation results as measured by the Jaccard index, our region‐based method still maintains a measurable advantage across the tested conditions. For our method, adding noise with a standard deviation of 4 resulted in a drop of 6.15% in the Jaccard index (from 0.76 to 0.71), whereas for the local Otsu method this drop was 13.33% (from 0.65 to 0.56). The largest difference in decrease of performance of the local Otsu method compared to ours was 21.63% when adding noise with a standard deviation of 8.

Table 3 gives an idea of the execution times of our implementation of the various algorithms tested when analysing a 1024 × 1024 greyscale image of nuclei running on an Intel i7 processor running at 3.3 MHz under the linux operating system to give an idea of the relative processing load. It is worth noting that our region‐based procedure depends not only on image size, image bit depth, number of regions detected (for the labelling step) but also involves additional operations (maximum projection and greyscale reconstruction). This does not compare favourably with other histogram thresholding methods that process, for example, a one‐dimensional histogram, but in terms of quality of results (Fig. 6), our regional method outperformed all others tested and has the advantage of not requiring postprocessing. On average across all other methods, the postprocessing overhead was 0.618 s, but this figure is likely to vary depending on the nature of the procedures applied and the number of regions and artefacts detected. Such postprocessing time also increases rapidly with image size, which affects the total overhead time required to achieve a desired result, particularly in large scale images. In this context, a high quality of the segmentation is certainly to be preferred over segmentation speed.

**Table 3:** Comparison of execution time (in seconds) of various thresholding methods.

Thresholding		Execution time	Requires
method	Type	(seconds)	postprocessing
OurRegional	Regional	6.056	No
L‐Otsu (18)	Local	3.167	Yes
OurGlobal	Global	2.278	Yes
L‐ Median (40)	Local	1.581	Yes
L‐Phansalkar (106)	Local	0.392	Yes
L‐Sauvola (98)	Local	0.333	Yes
L‐MidGrey (26)	Local	0.148	Yes
L‐Bernsen (22)	Local	0.140	Yes
L‐Contrast (14)	Local	0.089	Yes
L‐Niblack (18)	Local	0.052	Yes
L‐Mean(10)	Local	0.015	Yes
Huang	Global	0.013	Yes
Intermodes	Global	0.010	Yes
Percentile	Global	0.009	Yes
Shanbhag	Global	0.009	Yes
RenyiEntropy	Global	0.008	Yes
Yen	Global	0.008	Yes
Otsu	Global	0.007	Yes
Moments	Global	0.007	Yes
MaxEntropy	Global	0.007	Yes
Li	Global	0.007	Yes
Triangle	Global	0.007	Yes
Mean	Global	0.006	Yes
IsoData	Global	0.005	Yes
Minimum	Global	0.005	Yes

Note: The time figures sorted from slowest to fastest (smallest is best), indicate the average of 10 runs for processing an 8‐bit, 1024 × 1024 pixels image. The routines were coded as Java plugins for ImageJ, running on an Intel i7 processor at 3.3 MHz under the linux operating system (kernel version 3.16.7, Java 1.6.0).

In regard to memory constraints, the size of the stack *S* could become critical when dealing with large images. It is possible to use, instead, a cumulative image to store the maximum intensity region boundaries detected so far, while traversing the grey‐level space. Such modification is memory‐efficient, but our implementation was almost twice as slow due to the additional overhead required when creating and processing multiple single images in comparison with processing slices of a single stack. ImageJ implementations of the procedures described here (global and regional methods, the latter with both fast and memory‐efficient versions) as well as examples with sample images are available from http://www.mecourse.com/landinig/software/software.html

## Discussion

We present an alternative interpretation of Russ and Russ's observation on strategies used by operators to set image thresholds that enables automation of the search for optimal image threshold values. The method searches for global thresholds, but also provides a means to convert the analysis into a local, region‐based procedure that assesses the relation between candidate segmented regions and gradients in the original.

We compute a maximum projection of the average region boundary gradients obtained in threshold space to then find, region‐wise, threshold values at which maximum gradients exists in the original. By adding morphological constraints to the regions detected at all possible thresholds, gradient information is quantised and extracted by means of the regional maxima of the projection.

Image gradients are an important source of information for object identification. There appear to be physiological reasons for this, since the discovery of fields of neurons in cat and primate visual cortex, capable of detecting orientation patterns in images (Hubel & Wiesel, 1962, 1968). Based on this, a number of models of edge detection based on psychophysical techniques have also been suggested (Elder & Sachs, 2004). In terms of practical applications of computer vision techniques to imaging problems, the use of gradients has also been exploited (e.g. Chow & Kaneko, 1972; Hertz & Schafer, 1988). Nielsen *et al*. (2012) successfully used gradient information to verify perimeters and combine these with an active contours model for improving segmentation of nuclei in Feulgen stained sections. A different approach is used in seeded watershed segmentation (Beucher *et al*., 1979; Bengtsson *et al*., 2004) where gradients guide a space partition based on iteratively growing seeds. Although powerful, watershed segmentation sometimes suffers from oversegmentation arising from spurious seeds and from variability of the intensity of the gradients around objects, and in some respect, the strategy by operators in interactively adjusting threshold controls reminds us of the watershed ‘flooding’ process. One advantage of our approach, however, is the creation of the image projection (*P*), which results in a very simplified version of the image gradients from which optimally segmented objects can be identified via the computation of the regional maxima (something that cannot be achieved from the gradient image itself).

Other procedures that exploit multiple thresholds are *max‐trees* (Salambier *et al*., 1998) and *maximally stable extremal regions* (MSER) (Matas *et al*., 2002). Both approaches use threshold decomposition to generate a hierarchical data structure representing the relations of all thresholded regions of a greyscale image. Those relations are organised in graphs or trees containing large numbers of nodes (e.g. even in a relatively small image like Figure 1(A), threshold decomposition generates 27 622 dark and 28 648 bright regions with 39 378 and 40 363 boundaries, respectively) and this makes it difficult to represent and visualise in graph form or to be processed interactively (Tavares *et al*., 2015). MSER has been used for extraction of regions (extremal regions), which are stable (in terms of intensity or size) over a range of threshold values (Matas *et al*., 2002). This appears to be equivalent to Russ and Russ's observation of perimeters of regions changing slowly with changes in threshold setting. However, although stability is a key concept in MSER, it is not utilised in our regional threshold approach. Instead, circularity, size and gradient at the boundary of detected regions are the essential features for the detection of objects. For example, given low levels of background noise, faint ‘dark’ regions detected at a single greyscale level (i.e. one grey level below background) that pass the circularity and size criteria would have minimal stability in MSER terms, yet still can be successfully detected by our approach. Although it might be possible to implement criteria of size, circularity and gradient for the detection of regions using MSER or max‐trees structures (e.g. generate a tree, encode in it boundary gradients intensities and then search the tree for ‘stable’ regions), it is not necessary for our approach. We store in the stack *S* only those boundaries passing the specified size and circularity criteria. The subsequent maximal projection of the stack makes the selection of the boundary simpler, without having to identify stability features, since the boundary with the highest gradient (among all other boundaries corresponding to the same object at other greyscale levels) is retained in the maximum projection and can be extracted in a single pass by means of the regional maxima. The stack projection loses information about threshold level at which a boundary exists, but this information is not exploited in our approach, making the implementation simple in most imaging platforms using standard image processing procedures.

When compared to other common histogram‐based thresholding methods, our approach is computationally more demanding because of the brute force search over all possible threshold values and the additional processing required to extract the region boundaries, their gradients, the stack projection and the greyscale reconstruction to obtain the regional maxima. However, the region‐based method performance on our images of stained cell nuclei achieved better results than those obtained by the other methods.

Despite this, we have identified some situations where the region‐based method might not perform ideally. Noise in images is one such situation (although as shown earlier, this was not particular to our method only). In most bright field microscopy applications, noise is often not excessive because of well‐established means of circumventing it (for example by means of frame averaging). Another source of mis‐segmentation in the global version of the method is the presence of small, high‐contrast objects, which are not themselves target objects (because the high contrast objects result in high gradient boundaries). However, the region‐based version of the algorithm using object specification makes use of shape and size constraints that guarantee that the ‘correct’ regions remain in the analysis. Object specification, however, is a ‘double‐edge sword’; specifying objects with high circularity will probably result in rejecting groups of clumped nuclei early in the analysis (because a cluster of nuclei is more likely to have lower circularity and larger size than the individual nuclei that form it).

A final consideration with regards to the final binary fill described to generate regions from the detected boundaries is that although topologically simply connected objects (i.e. without holes, such as nuclei) might be segmented correctly with the region‐based approach presented, care might be necessary with objects having ‘child’ regions (i.e. regions located in holes of a parent region) and the use of binary filling routines that can preserve those.

Despite these caveats, our region‐based approach outperformed all the other methods tested here on the test set investigated. Although the method might not be applicable to any arbitrary image, it is worth considering for inclusion as an additional analytical tool for microscopy applications.
